# *Toxoplasma gondii* in Blood Donors: A Study in Boyer-Ahmad County, Southwest Iran

**DOI:** 10.1155/2018/3813612

**Published:** 2018-04-15

**Authors:** Abdolali Moshfe, Nasir Arefkhah, Bahador Sarkari, Saadat Kazemi, Ahmad Mardani

**Affiliations:** ^1^Cellular and Molecular Research Center, Yasuj University of Medical Sciences, Yasuj, Iran; ^2^Department of Parasitology and Mycology, School of Medicine, Shiraz University of Medical Sciences, Shiraz, Iran; ^3^Basic Sciences in Infectious Diseases Research Center, Shiraz University of Medical Sciences, Shiraz, Iran; ^4^Blood Transfusion Research Center, High Institute for Research and Education in Transfusion Medicine, Iranian Blood Transfusion Organization (IBTO), Tehran, Iran

## Abstract

*Toxoplasma gondii* is an important foodborne protozoan that can be transmitted through infected blood containing tachyzoite form of the parasite. The current study aimed to evaluate the prevalence of* T. gondii* infection and related risk factors among healthy blood donors in Boyer-Ahmad County, southwest Iran. Blood samples were taken from 285 healthy blood donors who voluntarily agreed to participate in this study. Sera and buffy coat were isolated from the blood samples for serological and molecular evaluations. The sera were tested for anti-*T. gondii *antibodies (both IgG and IgM), using a commercial ELISA kit. The buffy coat of seropositive cases was evaluated for detection of* T. gondii *DNA by PCR. Moreover, a structured questionnaire, containing socioepidemiological data and possible risk factors, was filled out by each participant during sample collection. Anti-*T. gondii* antibodies were detected in sera of 48/285 (16.8%) participants. Only two of the subjects (0.7%) were seropositive for both IgG and IgM antibodies.* T. gondii *DNA was not detected in buffy coat of any of the seropositive cases. Risk factors such as contact with soil (OR, 9.7; 95% CI, 4.9–19.4) and consumption of semicooked meat (OR, 2.5; 95% CI, 1.2–5.03) were statistically associated with seropositivity to* T. gondii*. The seroprevalence rate of* T. gondii* antibodies in the blood donors of Boyer-Ahmad County was not high in comparison with other regions in Iran. In this study, consumption of undercooked meats, job, and contact with soil were independent risk factors associated with* T. gondii *infection, which can be considered as potential sources of* T*.* gondii* infection.

## 1. Introduction

*Toxoplasma gondii *is often a water and foodborne pathogen with a wide and considerable distribution which can be transmitted through blood transfusion and causes acute severe complication such as encephalitis in immunocompromised blood recipients [1, 2]. Toxoplasmosis may result in abortion or neurological abnormalities in human fetus following intrauterine transmission [1, 3]. Toxoplasmosis is an asymptomatic mild infection in most of the immunocompetent individuals, yet a number of studies have shown a significant association between latent toxoplasmosis and psychiatric disorder among immunocompetent individuals [4–6].

The main routes of* T. gondii* infection in human are ingesting of sporulated oocysts in contaminated water, vegetables, and fruits along with eating undercooked contaminated meat and also vertical transmission at the time of pregnancy. Moreover, the healthy human can be infected through blood transfusion or organ transplantation. Toxoplasmosis is a common infection in human and animals in all areas of Iran [2, 7–11]. Blood donors, especially those who are in the acute phase of the infection, can impose risk of* T. gondii* infection for the susceptible recipients [12, 13]. The mean prevalence of toxoplasmosis among Iranian blood donors varied between 12.3% and 52.8% [14]. Recent studies reported anti-*T. gondii* IgG antibodies in sera of 19.3% of blood donors, 8.5% of female university students, and 8.9% of pregnant women in southern Iran [2, 15, 16].

The current study was performed to assess the seroprevalence rate of* T. gondii* and possible associated risk factors among blood donors in Boyer-Ahmad in southwest Iran.

## 2. Materials and Methods

### 2.1. Study Area

This cross-sectional study was conducted on healthy blood donors of Boyer-Ahmad county, located in southwest Iran, from April to August 2015. Boyer-Ahmad is located in a mountainous and cold region of Iran. Snow and rainfall are plentiful in this region, especially in fall and winter seasons. The mean altitude in the area where the Boyer-Ahmad people live is 1800–2200 m above the sea level. The area has geographical coordinates between latitudes 30–9° and 31–27°N and between longitudes 49–55° and 51–42°E. Animal husbandry and gardening are common in this district. Food habits of more people are composed of meat, milk, and local vegetables. Parasitic diseases are not uncommon in this area and the area is considered as a focus of human fascioliasis as well as visceral leishmaniasis [17–21]. Also, high levels of blood-borne diseases including hepatitis C and B have been reported from the area [22, 23].

### 2.2. Sampling

Subjects of this study were recruited from healthy blood donors who knowingly agreed for participation in the study. All participating individuals were given a questionnaire before collection of blood sample, which provided information about their age, sex, residence area, level of education, occupation, and risk factors including the consumption of raw/undercooked meat, contact with cat, contact with soil in gardening or agricultural activities, and history of blood transfusion. Awareness about toxoplasmosis and clinical symptoms including fever, rash, and lymphadenopathy were also recorded. 5 mL of fresh blood was collected from each blood donor in plastic K_2_EDTA-containing tubes. Samples were centrifuged and buffy coat and sera were isolated from each sample. Collected samples were kept at −20°C until use.

### 2.3. Serologic Testing

All of the collected sera were tested for detection of anti-*T. gondii* IgG and IgM antibodies, using a commercial ELISA kit (ACON Biotech, Hangzhou, China), based on the manufacturer's instructions. Index value was obtained for both IgG and IgM. An index value ≤ 0.9 IU/mL was regarded as negative result, while the equivocal range was defined between 0.9 and 1.1 IU/mL and index value greater than 1.1 IU/mL was considered as positive result for both IgG and IgM.

### 2.4. DNA Extraction and PCR Amplification

DNA was isolated from the buffy coat of each sample, using the phenol-chloroform extraction method as previously described [2]. The conventional PCR for detection of* T. gondii DNA *was performed targeting a 529 bp gene, with primers TOXOF (5′- CAGGGAGGAAGACGAAAGTTG- 3′) and TOXOR (5′- CAGACACAGTGCATCTGGATT-3′) [24]. The total reaction volume was 25 *μ*L, containing 1 unit of Taq polymerase, 12.5 *μ*L of 2x Master Mix Red, 1.5 mM of MgCl_2_, 1 *μ*L of each 20-picomole primer, 50 ng of extracted DNA, and the remaining nuclease-free water. The PCR program was set for 5 minutes at 94°C before cycling, followed by 30 cycles of denaturation at 94°C for 35 seconds, annealing at 56°C for 1 minute, extension at 72°C for 1 minute, a final extension at 72°C for 10 minutes, and final hold at 4°C for 10 minutes. Amplification was performed with negative along with positive control of* T. gondii* (a kind gift of Dr. Q. Asgari). The 529 bp PCR products were separated by electrophoresis in 1.5% agarose gel and stained with ethidium bromide.

### 2.5. Statistical Analysis

SPSS 18 software was used for all statistical analyses. The frequency of dependent variables was described, using descriptive statistics, and chi-squared and regression logistical tests (*p* < 0.05) were used to find out any possible association between qualitative variables and seropositivity to toxoplasmosis.

## 3. Results

### 3.1. Demographic Features of the Participants

Overall, 285 healthy blood donors were recruited in this study. The mean age of the subjects was 37 (±9.53) years. Most of the blood donors (41.8%) were in the age group of 28–37 years. The majority of the subjects were male (96.8%). Most of the subjects (36.5%) were employees.

### 3.2. Seroprevalence of Anti-*T. gondii* Antibodies

Anti-*T. gondii *antibodies were detected in sera of 48 out of 285 blood donors. Of these, 46 cases (16.30%) were seropositive only for IgG and 2 cases were seropositive for both IgG and IgM. Seropositivity in males and females was 16.3% and 11.1%, respectively.

### 3.3. Risk Factors for* T. gondii* Seropositivity

In the univariate analysis, three variables including contact with soil (*p* = 0.01), consumption of undercooked meat (as barbecue or kebab), (*p* = 0.007), and job (*p* = 0.040), were documented as associated risk factors for* T. gondii* seropositivity. Other risk factors and also demographic features of the blood donors were not statistically associated with the acquisition of* T. gondii* infection (*p* ≥ 0.05) (Table 1).

### 3.4. Detection of* T. gondii* DNA in Seropositive Subjects

None of the 48 seropositive subjects were positive for* T. gondii* by molecular (PCR) method.

## 4. Discussion

This is a cross-sectional study regarding the seroprevalence and molecular evaluation of* T. gondii* infection among healthy blood donors of Boyer-Ahmad County in southwest Iran. The overall seroprevalence of 16.8% for toxoplasmosis in the current study indicates a considerable rate of toxoplasmosis in the population, but the seroprevalence rate of toxoplasmosis in our study is lower than the rates reported in blood donors from most of the areas in Iran [2, 7, 25] and other regions as reported from Turkey (22.59%) [26], Czech Republic (34.23%) [27], Brazil (60%) [28], Saudi Arabia (40%) [29], Iraq (32.75%) [30], Egypt (59.6%) [31], and India (53.7%) [32]. Variations in the rate of seropositivity of* T. gondii* in different regions of the world or in different areas of a given country can be attributed to the differences in climate, topographical conditions, and food behavior.

Lower seroprevalence of toxoplasmosis in high altitude and cold climates has been reported, as* T. gondii* oocysts cannot survive for a long time in such environmental conditions. In the present study, consumption of undercooked meat was identified as a risk factor related to* T. gondii* seropositivity in blood donors. These findings indicate that the ingestion of undercooked meat, mainly sheep and goat, containing tissue cysts might be one of the main sources of* T. gondii *infection in this area [33, 34]. This notion has already been documented in other areas of Iran [8, 10]. Exposure to the soil, followed by oocysts in soil, is an important risk factor for acquisition of* T. gondii.* Risk factors of toxoplasmosis vary in different geographic regions. Contact with cats in Mexico and eating raw shellfish as well as exposure to domestic cats in Taiwan [35] have been counted as the main risk factors for* T. gondii* infection among the blood donors. In rural communities of northern Iran, consumption of undercooked sheep and goat meat and unwashed raw vegetables or fruits have been considered as the main risk factors for* T. gondii* infection [34], while contact with cats and consuming raw vegetables and raw milk/egg were identified as independent risk factors for* T. gondii *seropositivity among the healthy blood donors in the southeast of the country [36].

In the current study, there was a significant correlation between job and seropositivity with* T*.* gondii*. This fact can be justified as most of the people in the studied area live on animal husbandry and agricultural activities, which increases the chance of* T. gondii* infection. None of the seropositive cases were positive for* T. gondii* DNA. This indicates that all of the seropositive cases have been in the chronic phase of toxoplasmosis and have no risk of* T. gondii* infection for the recipients.

This study showed that more than 16% of the healthy blood donors in Boyer-Ahmad County in southwest Iran have anti-*T. gondii* antibodies in their sera and the consumption of undercooked meats, job, and contact with soil were three independent risk factors associated with* T. gondii *infection.

## Figures and Tables

**Table 1:** Risk factors analysis of seropositivity to toxoplasmosis among blood donors in southwest Iran.

Variables	Frequency (number)	Seropositivity (%)	Odds ratio (95% confidence interval)	*p* value
*Blood transfusion*				
Yes	8	37.5	3.09 (0.714–13.407)	0.135
No	277	16.2	1	
*Place of residence*				0.602
City	203	17.5	1.257 (0.618–2.559)	
Village	82	14.6	1	
*Eating of semicooked meat*				0.007
Yes	157	22.30	2.538 (1.278–5.038)	
No	128	10.15	1	
*Contact with cat*				0.841
Yes	51	15.6	0.902 (0.394–2.065)	
No	234	20.51		
*Taking immunosuppressive drugs*				0.199
Yes	5	40	3.391 (.551–20.866)	
No	280	16-4		
*Contact with soil*				0.001
Yes	61	47.5	9.778 (4.914–19.457)	
No	224	8.5		
*Job*				0.040
Employees	112	25	1	
Other business	81	16	1.744	
Student	24	4	7.667	
Farmer and rancher	55	9.09	3.333	
Unemployed	13	7.70	4.000	
*Educational level*				0.592
Secondary level and below	56	17.86	1.049 (0.469–2.348)	
High-school level	89	13.5	1.463 (0.696–3.075)	
University level	140	18.6	1	
*Blood group *				0.285
A	88	13.6	1.715 (0.812–3.621)	
B	57	15.8	1.444 (0.628–3.324)	
AB	18	5.5	4.604 (0.585–36.226)	
O	122	21.3	1	
*Washing vegetables with antiseptic materials*				0.587
Yes	73	19.7	1	
No	212	16.03	1.242 (.624–2.473)	
*Awareness about toxoplasmosis*				0.702
Yes	10	10	0.539 (0.067–4.357)	
No	275	17.1	1	
